# The influence of technostress, work–family conflict, and perceived organisational support on workplace flourishing amidst COVID-19

**DOI:** 10.3389/fpsyg.2022.921211

**Published:** 2022-07-26

**Authors:** Martha Harunavamwe, Chené Ward

**Affiliations:** ^1^Department of Human Resource Management, University of Pretoria, Pretoria, South Africa; ^2^Department of Industrial Psychology, University of the Free State, Bloemfontein, South Africa

**Keywords:** technostress, work-family conflict, perceived organisational support, workplace flourishing, COVID-19, remote working

## Abstract

The remote working environment is characterised by excessive use of new technology and work activities that extend to personal time. It is expected of each employee to balance multiple roles whilst maintaining maximum performance and individual wellbeing; however, without adequate support from an organisation, employees languish instead of flourish. The current study applied a model to investigate the combined effect of technostress, work–family conflict, and perceived organisational support on workplace flourishing for higher education employees. The study followed a cross-sectional quantitative research framework. Data were collected from a sample of 227 academic and support staff employees from a selected residential University in South Africa. The results indicated that technostress through perceived organisational support and through work–family conflict influences workplace flourishing. No direct significant effect was reported between technostress and workplace flourishing. Technostress, work–family conflict, and perceived organisational support combined explained 47% variance in workplace flourishing. Perceived organisational support displayed the strongest direct effect on workplace flourishing, and technostress is a strong determinant of work–family conflict, which then mediates the relationship between technostress and workplace flourishing. The study concluded that providing organisational support and creating policies favourable to work–life balance assist employees in managing techno-overload, techno-invasion, and techno-complexity (technostress) better and enhance workplace flourishing. Although employees struggle in the remote working context with demands imposed by techno-overload, techno-invasion, and techno-complexity, the results indicate that perceived organisational support and balanced work life act as job resources that enhance emotional, psychological, and subjective wellbeing (workplace flourishing).

## Introduction

Higher education employees have been exposed to new changes, shifting from face-to-face classes to the emergency of online platforms in the middle of a stressful pandemic. Some negative consequences have been observed, particularly for workers' wellbeing and productivity. A deep comprehension on how individuals experienced remote working supported by technologies has become very crucial (Lades et al., 2020). In such conditions, positive academic and support staff functioning is an important objective desired by every leader. It is relevant for researchers to unpack the progressive aspects related to positive academic practices. Accordingly, particular consideration should be given to constructs, such as workplace flourishing, which encompass all aspects of wellbeing. This study examines emotional, psychological, and subjective wellbeing (workplace flourishing) amongst employees at a residential higher education institution in South Africa during the COVID-19 lockdown, which was characterised by remote working, social distancing, and measures that transformed the working practices of most employees across the country. A number of studies have identified the extensive challenges imposed by the pandemic in the workplace, especially in terms of wellbeing (Jemberie et al., 2020; Patrick et al., 2020; Sibley et al., 2020). Recently, Wanberg et al. (2020) have discovered that higher education employees experience and display high depressive symptoms coupled with an increase in life dissatisfaction due to work pressure imposed by the pandemic. Contrary to these findings, some studies noted that most of the academics who are familiar with online learning tools barely experienced stress or anxiety during the shift from face-to-face classes to online learning platforms during the pandemic (Apouey et al., 2020; Spagnoli et al., 2020). The conflicting findings simply suggest how different employees' wellbeing was impacted by the pandemic and the change of the work scenario; hence, these conflicting findings call for clarity.

COVID-19 penetrated social, cultural, and technological barricades that blocked virtual working leading to a structural shift in where work occurs (Lund et al., 2020). Remote working created stress, which was exacerbated by the usage of information and communication technologies (ICT). ICT prompted work intensification, which consequently produced negative outcomes, including strain, anxiety, tension, and discomfort, which then led to poor work performance, increased work–family conflict, and emotional exhaustion (Penado Abilleira et al., 2021). Hence, the adaptation of ICT came with both benefits and challenges. ICT altered the work environment, the culture, and the means by which employees performed their given work. The greater reliance on ICT has also bred work extension expectations, with most employees being always accessible, working at a higher speed, and producing better results, but experiencing overload and strain (Spagnoli et al., 2020). This has resulted in technology being considered as a fundamental part of major organisational functions, as well as a source of stress (Le Roux and Botha, 2021). Exploring technostress has, therefore, become pertinent and relevant.

Amidst COVID-19, institutions of higher learning had to reconsider how current technology could allow business to function as usual. Academics and support staff had to frequently interact with technology, and the continuous upgrading of the online systems and software exposed them to constant strain, considering that they do not always have the knowledge required to use new and updated technologies (Li and Wang, 2021). However, lockdown left academics improvising new forms of teaching, resembling emergency remote teaching (Dey et al., 2020), which required the ability to integrate technology into lectures. For some, this increased ambiguity and burnout due to the complex nature of technology (Schildkamp et al., 2020). Thus, the virtual work arrangements imposed by the COVID-19 crisis have increased workload and the levels of technostress, which eventually threatens wellbeing (Spagnoli et al., 2020).

Technology has the capacity to upgrade the work environment and enhance performance, productivity, and efficiency and create flexibility; however, it can also result in adverse consequences for individuals' physical, psychological, and cognitive wellbeing. The negative outcomes of technostress subsequently affect the organisations through low productivity caused by decreased employee wellbeing, low satisfaction, lack of commitment, and possible burnout and languishing (Salanova, 2020). With proper support from the organisations, technology can improve workplace efficiency and productivity; however without such perceived support, technology tends to be a burden and a source of strain. Thus, when employees perceive that their supervisors and the organisation at large are supportive and fulfil their socio-emotional needs, they are more likely to flourish in their work. Accordingly, workplace flourishing is further intensified in the presence of significant resources that are associated with a job, including perceived organisational support (POS). Within the premises of the job demands–resources model (JD–R model), technostress creators can be regarded as job demands (including techno-invasion, techno-complexity, and techno-overload) that can be curbed by the supply of adequate job and personal resources, such as organisational support and work–life balance. Putranto et al. (2021) added that, when experiencing technostress, POS creates a feeling of security and satisfaction of employees' emotional needs to positive effect. This implies that employees who regard their organisations as supportive are more likely to experience positive psychological wellbeing even when they experience challenges, enabling them to flourish.

Within the remote settings, roles of employees have doubled or tripled, resulting in the interference between work and family (Le Roux and Botha, 2021). Since restrictive lockdowns were imposed, employees were confined to their homes with their children and partners. This meant that, whilst one was considered to be working, simultaneously, it included doing house chores, taking care of the children, and providing homeschooling, whilst still dealing with the reality of the COVID-19 disease (Spagnoli et al., 2020). All of this contributed to the experience of the crisis of work–life balance (Tomohiro, 2021). Based on the sentiments of Tomohiro (2021), a flexible work style and persistent confinement with technology may lead to a decrease in employee wellbeing and interfere with employees' private life by blurring working hours and non-working hours. However, with adequate support from the supervisor and the organisation at large, employees experience emotional security and engagement. A recent study has noted that, without support from an organisation, the changes brought by digitalisation, where academics are expected to adapt to the high demands imposed on them, threaten their mental and physical resources, thereby depreciating their wellbeing (Penado Abilleira et al., 2021). The impact of adaption in the current work context has largely remained untested, more so in residential higher education institutions. A relative burden has been placed on employees' work–life balance with increased adaption to technology as a tool to work from home, and adequate support needs to be provided to ensure employee flourishing. This study seeks to establish the combined effect of technostress, work–family conflict, and perceived organisational support on workplace flourishing in higher education.

## Literature review

### Workplace flourishing

The concept of workplace flourishing is regarded as the most prominent multidimensional construct for the wellbeing models (Seligman, 2012). It encompasses emotional, psychological, and subjective wellbeing in one context, and the construct provides an indication of the way employees feel and function in an organisation (Rothmann, 2014). As a multifaceted approach to wellbeing, workplace flourishing considers the extent to which one experiences a purposeful and meaningful life at work (Redelinghuys et al., 2019), being engaged, interested, and competent in one's work; feeling self-respect and optimism; being respected by others; having supportive relationships; and socially contributing to the happiness of others (Diener et al., 2010). Workplace flourishing is defined as the employees' state of wellbeing, which is a result of frequent positive experiences and favourable job-related experiences. The concept has become relevant due to challenges experienced by most employees in trying to adjust to the new nature of work (Rautenbach, 2015). According to Huppert and So (2009), when an individual successfully thrives and experiences a sense of wellbeing in almost all areas of their work life, it is summed up as flourishing at work. This implies that one functions effectively and perceives life as going really well; thus, wellbeing is fully conceptualised in those lenses. Flourishing, in general, is seen as a time when an employee experiences high levels of personal wellbeing, coupled with minimum pain, pleasant emotions, and being involved in interesting activities, which brings overall satisfaction with life (Diener et al., 2010).

Given that emotional, psychological, and subjective wellbeing are the collective components that explain flourishing and languishing of employees, an individual's level of flourishing or languishing can be evaluated on the mental health continuum (MHC) developed by Keyes and Annas (2009). According to this continuum, flourishing is a state in which individuals experience high levels of emotional, psychological, and subjective wellbeing; on the other hand, languishing is a state in which individuals do not have much good feeling toward life, and they also do not see themselves as functioning well in life (Keyes and Annas, 2009). In line with the above, according to the JD–R model, employees who sense that their job demands surpass the available resources because of complexity or emotional, psychological, or physical strain will feel incapable to cope with management at work (May et al., 2004; Schaufeli and Bakker, 2004). This can potentially cause employees to feel that they are not in control of their environment, and, consequently, they experience burnout and languish rather than flourish. Exposure to technostress and persistent work–family conflict involves an inescapable component of strain, as employees experience a variety of complexities and multiple job demands; hence, technostress creators and work–family conflict are considered as strenuous job demands. In terms of the job demands–resources (JD–R) model (Demerouti et al., 2001), although employees face stringent demands, they also have various personal and job resources that may well counter the influences of the demands. Employees need an incrementation of resources to restore a balance between job demands and resources. In this case, more ICT support and more favourable work–life balance strategies/policies may act as resources that may inhibit technostress and work–family conflict and facilitate flourishing despite the challenges facing employees.

### Technostress

Knani (2013) states that technostress emanates from the excessive utilisation of ICTs, including laptops, cellphones, constant instant messaging, e-mail, and voice mail. The experience of technostress is attributed to one's attempt to handle the constantly changing and developing ICTs, which pose new challenges in adjusting to the frequently changing physical, social, and cognitive demands posed by ICT in the work settings. The use of ICT can create difficulties for employees through generating a variety of stressors, such as overload, invasion of family time, role ambiguity, complexity, and job insecurity. Tarafdar et al. (2007) conceptualised the technostress phenomenon as a form of stress that is experienced by end users in organisations as a result of operating the ICTs. Atanasoff and Venable (2017) defined technostress as a mental stress created by use of technology, resulting in strong emotional responses associated with fear and anxiety. Ragu-Nathan et al. (2008) noted that technostress consists of five components, namely, techno-complexity, techno-overload, techno-insecurity, techno-invasion, and techno-uncertainty. These dimensions can be used to measure levels of technostress experienced by employees in organisations. Techno-overload describes an increase in the rate and amount of work, which causes employees to execute their duties at a high speed and spend more time on work (Tarafdar et al., 2007). The volume of information that employees have to absorb from ICTs can be beyond comprehension, such that it results in negative outcomes and detrimental effects on individual health. For instance, in the university setting, frequently alternating between diverse devices (laptops, phones, emails, and instant messaging), as well as performing diverse job tasks, reduces task quality, and efficiency as the employee's mind requires an adequate time span to process the absorbed information (Ingusci et al., 2021). Techno-invasion is the pervasive invasion of an employee's personal life by ICTs, therefore blurring the boundaries between work and private life (La Torre et al., 2020). Techno-invasion has a direct impact on work–life balance. Mahapatra and Pati (2018) found techno-invasion to be negatively related to wellbeing in employees. Techno-complexity is the high complexities of new ICTs, which cause an employee to feel incompetent (Barber and Santuzzi, 2015). More complex devices and software evoke more frustration and demoralise individuals as they try to understand how the device works. This negatively impacts an organisation, because, when employees are frustrated and demoralised, their performance and productivity decrease. When technology is perceived to be too complex to carry out a task, or to incorporate into work, an employee may experience the techno-stressor called techno-complexity, which negatively affects performance and wellbeing (Day et al., 2012). Techno-insecurity considers what employees experience when they fear that they may lose their jobs and be replaced by new information systems or by better equipped or more technologically skilled employees (Ibrahim and Yusoff, 2015). Thus, instead of focusing on producing good results, employees spend most of their time frequently experiencing fear of job loss due to automation.

Previous studies have concluded that people reporting elevated levels of technostress are more likely to suffer the psychological strains of diminished commitment, struggle to flourish and display signs of languishing (Tarafdar and Stich, 2021), have poor self-esteem (Korzynski et al., 2021), and dissatisfaction with the IT system (Tams et al., 2020), harmful psychological responses, and burnout (Afifi et al., 2018), and their wellbeing is negatively affected. It is, therefore, proposed that technostress has a negative influence on workplace flourishing.

### Work–family conflict

Work–family conflict is derived from the work–life balance construct that is defined as the link between an individual's work and life; when the balance is achieved, no interference is seen between an employee's family life and his or her work life (Muthukumar et al., 2014). It is thus the relationship between work and non-work aspects of an employee's life (Kelliher, 2016). Achieving a satisfactory balance may, however, mean restricting one side, normally the work side to create more time for family. A study by Haar et al. (2014) discovered that employees who master how to balance family and work life experience more satisfaction in their life, and this positively impacts their mental and physical health. The construct of work–family conflict (WFC) is made up of three components, namely, the behaviour-based conflict, the strain-based conflict, and the time-based conflict (Kossek and Lee, 2017). Behaviour-based conflict refers to situations where certain behaviours, rules, and expectations required by one role (work or family) are found to be incompatible with those required for the other role (Loscalzo et al., 2019). Time-based conflict refers to the amount of time needed by one of the two roles (i.e., work–family or family–work) that prevents the possibility of fulfilling the other role's expectations (Loscalzo et al., 2019). Lastly, strain-based conflict is experienced when an individual is strained and fatigued and experiences tension, anxiety, and dissatisfaction in one domain, which then negatively influences his or her performance in the other domain (Kossek and Lee, 2017).

Work–family conflict directly and together with technostress may negatively influence workplace flourishing. Technostress creators (techno-invasion) are associated with work–family conflict, behavioural stress, and ICTs, and support the negative spillover between work life and family life (Kelliher, 2016). When ICT deeply penetrates the family boundaries (i.e., high techno-invasion), the individual will have less time and energy to devote to his/her family responsibilities, resulting in frustrations, constant feelings of failure, and a negative influence on flourishing (Salo et al., 2019).

### Perceived organisational support

Karim et al. (2019) defined perceived organisational support as the perception of an individual pertaining to the extent to which his or her organisation looks after his or her wellbeing and values his or her contribution. POS is a multidimensional construct. The first dimension is fairness in organisational procedures: this is derived from the theory of organisational justice, which uses fair procedures to determine the allocation of resources. Employees regard these procedures as essential to their long-term interests and wellbeing (Jabagi et al., 2020). The second dimension is supervisor support. Supervisors are regarded as the agents of an organisation and have a close relationship with top management; therefore, employees regard supervisor support as organisational support (Jabagi et al., 2020). Organisational rewards and working conditions make up the last dimension. This refers to human resource practices that take employees' contributions, their working conditions, and characteristics of their job into consideration. In the context of this study, perceived organisational support is regarded as a job resource that may assist individuals with challenges emanating from the use of technology and failure to balance work life and family life due to work demands. Applying the organisational support theory (OST), when employees assume that their organisations provide them with intangible and tangible support, a norm of reciprocity creates a feeling of obligation amongst employees that drive them to help their organisations to achieve their goals. A reciprocity norm recommends that employees with high POS pay off their organisations in the form of flourishing and by engaging in their work (Fredrickson and Losada, 2005; Karim et al., 2019). Therefore, perceived organisational support directly and indirectly influences flourishing.

### Theoretical framework [the job demands–resources model (JD–R model)]

The JD–R model is rooted in the premise that certain aspects of a job or specific field are deemed too demanding by an individual, causing him or her excessive stress and overtaxing, which result in exhaustion and languishing (Demerouti and Bakker, 2011). The model focuses on the interaction between job resources and job demands and how the interaction results in health impairment, such as languishing, or impacts motivation, such as employee engagement (Demerouti et al., 2001). Job demands encompass any social, physical, or organisational aspects of work that requires an employee to dedicate his or her mental or physical effort. Job demands are associated with certain psychological or physiological costs (Llorens et al., 2006). These include things like unusually high work pressure, irregular working hours (interfering with work–life balance), or a poor work environment (Demerouti and Bakker, 2011). Job resources include organisational, physical, and social aspects of the job that enables individuals to manage and take control of their job demands, achieve work-related goals, and reduce stress, as well as stimulate growth and development (Llorens et al., 2006).

The current study views technostress as a job demand, which, if not managed, may negatively influence work–life balance. It is thus expected that both technostressors and work–family conflict will negatively influence workplace flourishing (La Torre et al., 2020). Techno-invasion forces employees to handle a wide variety of work demands during family time at home. This situation reduces the employees' ability to be fully absorbed and enjoy what they wish to do at home and, consequently, has a negative effect at home (Mahapatra and Pati, 2018). Techno-complexity coerces individuals to spend much of their time and cognitive effort trying to learn and master the application of different technologies in their jobs. It requires individuals to continually develop their skills to keep up with new tools. This process can possibly negatively impact employees' effectiveness in both work and life roles, since more time is invested in training; this, in turn, causes anxiety and affects individuals' emotional and psychological wellbeing (Karim et al., 2019). Thus, the combined effects of technostress and work–family conflict may be detrimental to employee health and wellbeing, and this becomes worse in remote settings. Fortunately, Putranto et al. (2021) indicated that, in such work contexts, POS is seen as a job resource that lessens stress and supports and creates a feeling of security and satisfaction of the employees' psychological and emotional needs for positive effect. Thus, individuals who perceive their supervisors and organisations as supportive have a greater chance of experiencing positive psychological wellbeing, and, despite the challenges they encounter, they are more likely to flourish than to languish (Mahapatra and Pati, 2018).

Supportive organisations and leaders monitor the signs and effects of technostress and immediately provide corrective measures and good practices, particularly during times of crisis when employees are expected to meet certain targets (Putranto et al., 2021). The introduction of good practices relating to the use of technology, such as compulsory training in new devices, systems and software, use of a single device at a time, and disconnection (during non-work times), is an achievable preventive intervention that individuals in supportive environments are encouraged to implement. Therefore, POS may assist individuals in coping with demands relating to technostress and conditions leading to work–family conflict. The JD–R model suggests that excessive job demands result in the depletion of employees' personal and job resources and energy, which could result in burnout and health deterioration; thus, one would languish instead of flourish in the workplace (Hakanen et al., 2008).

Individuals who experience flourishing are emotionally, cognitively, and physically fit compared to those that are languishing (Jemberie et al., 2020). Flourishing individuals are creative and experience less helplessness and more favourable emotions; they achieve higher and produce more positive outcomes in the work context; hence, they benefit an organisation more compared to those that experience adverse emotions (Patrick et al., 2020). With all these benefits of flourishing in mind, the current study examined the indirect and direct effects of technostress, work–family conflict, and POS on workplace flourishing. The idea is to develop a model to assist employees working in remote contexts to identify job resources that will enable them to manage the job demands imposed by technostress creators as well as to counteract work–family conflict. The study positioned POS as an external job resource that counteracts the demands imposed by both technostress creators and work–family conflict, and this, in turn, helps to sustain a continuous positive emotional and psychological state of employees leading to workplace flourishing. The following four propositions guided this exploratory study: (1) Technostress, work–family conflict, and POS have a direct influence on workplace flourishing; (2) POS mediates the link between technostress and workplace flourishing; (3) Work–family conflict mediates the relationship between technostress and workplace flourishing; (4) Both POS and work–family conflict mediate the relationship between technostress and workplace flourishing.

## Methods

The study applied a quantitative research framework. This design was adopted and found appropriate due to its systematic and scientific nature of investigating data and their relationships. The approach has been successfully followed in studies of a similar nature (Redelinghuys et al., 2019). The study aimed to test the propositions and describe relationships between four variables (workplace flourishing as the dependent variable and three independent variables: technostress, POS, and work–life balance).

### Sample of participants

A survey was conducted with the employees at a selected residential University in South Africa. Data were collected through online platforms using evasys, and the sample was made up of both academic and support staff. The participants completed a cross-sectional survey, utilising a self-reported questionnaire. A total of 227 employees completed the survey. The participants completed the survey when lockdown measures were still in place. Amongst the participants, the majority (68%) were female; in terms of age, the majority were between the ages of 31 and 40 years (38%), whilst the minority were above 60 years (6%). Most of the participants, 62%, were academic staff, whilst 38% were support staff.

### Measures

#### Flourishing at work

The flourishing at work construct was assessed using the Flourishing-at-Work Scale (FAW). The scale was developed and validated by Diener et al. (2010). It consists of items measuring emotional wellbeing (7 items), psychological wellbeing (7 items), and subjective wellbeing (7 items). The scale is a self-report instrument that includes three subscales as noted above. In the current study, permission to use the adapted scale was sought from and granted by Redelinghuys et al. (2019). The response scale is scored on a five-point Likert scale, varying between poles of intensity from 1 (strongly disagree) to 5 (strongly agree). A higher response aggregate indicates higher levels of workplace flourishing, and a lower response aggregate indicates otherwise. Evidence of both construct validity and internal consistency reliability has been established by Di Fabio et al. (2017) with the respective scores α = 0.88 and α = 0.83. The current study also observed an acceptable internal consistency for the Flourishing-at-Work Scale (α 265 = 0.957).

#### Technostress questionnaire

The technostress variable was assessed using the Technostress Questionnaire, which is made up of the five dimensions of technostress noted in the literature review section (Tarafdar et al., 2007). The scale applied in this study consists of 23 items that are assessed on a 5-point Likert scale, with 5 indicating “strongly agree” and 1 indicating “strongly disagree.” According to Tarafdar and Stich (2021), the scale is reliable with the Cronbach's alpha for all the dimensions above 0.80, i.e., techno-invasion, 0.81; techno-overload, 0.89; techno-complexity, 0.84; techno-uncertainty, 0.82; and techno-insecurity, 0.84. The current study obtained an acceptable internal consistency for the technostress questionnaire (α = 0.881).

#### Work–family conflict scale

Work–family conflict was assessed through the Work–Family Conflict Scale (WFC) developed by Chen et al. (2021). The entire scale contains a total of 18 items. According to Carlson et al. (2000), the three-dimensional scale consists of three sections, including strain-based conflict, behaviour-based conflict, as well as time-based conflict. The internal reliability estimates for the Work–Family Conflict Scale measure was found acceptable in previous studies, ranging from 0.84 to 0.94 (Brough et al., 2014). The Work–Family Conflict Scale has discriminant validity (Chen et al., 2021), and it has been proved to be an accurate measure to assess the level of work–family conflict. Consistent with the above, the current study observed an acceptable internal consistency (α = 0.928).

#### Perceived organisational support scale

As far back as 1986, Eisenberger et al. (1986) developed the POS questionnaire. The original 36-item scale measures POS and its sub-dimensions (Wojtkowska et al., 2016). However, the current study used the shortened version, which is made up of 8 items. The questionnaire uses a 7-point Likert scale where 7 represents “strongly agree” and 1 represents “strongly disagree.” The scale has an internal consistency of 0.952 in the study by Wojtkowska et al. (2016), and the Cronbach's alpha was 0.88 in a study by Hinschberger (2009). The current study observed an acceptable internal consistency for the Perceived Organisational Support Scale (α = 0.900).

### Research procedure

The respondents were recruited from a selected university in South Africa. Ethical clearance was applied for and granted by the University of the Free State, specifically the Economic Management Sciences Research Ethics Committee (GHREC), with reference No. HSD2021/1827/21. After obtaining the permission, questionnaires were distributed *via* online platforms. The questionnaire included a clause for voluntary participation and the guarantee for both anonymity and confidentiality.

### Analytical procedure

Preliminary data analysis was done using Statistical Package for the Social Sciences, SPSS version 28. This included all the descriptive statistics and the Cronbach's alpha reliability tests. All measures were then subjected to confirmatory factor analysis, which was conducted using Lisrel 10.3. This was used to determine the psychometric properties of the measures used in the study, using the goodness-of-fit statistics, including standardised root mean square residual (SRMR), root mean square error of approximation (RMSEA), and comparative fit index (CFI). To address the objectives of the study and to evaluate the different propositions, the variance-based structural equation modelling was utilised.

The proposed model was tested following a two-step process as instructed by Henseler et al. (2009). In this process, the outer model, which is the measurement model, was evaluated first to assess the relevant quality criteria. The main purpose of the measurement model is to establish whether the measurements applied to operationalise the latent variables (i.e., technostress, workplace flourishing, and work–family conflict) are reliable and valid. The quality criteria associated with an acceptable outer model include (1) internal consistency, which is evaluated through composite reliability scores, which should be 0.7 and higher, (2) the discriminant validity assessed through the heterotrait-monotrait (HTMT) ratio values, which should be lower than 0.9, (3) the convergent validity, which is assessed through average variance extracted (AVE) values, which should be 0.5 and higher, and (4) the indicators (i.e., dimensions of constructs), which should have significant loadings on their respective constructs (Hair et al., 2019). Subsequently, the inner model, which is the structural model, was evaluated through determining the size of the path coefficients using the beta values, assessing the significance levels of the path coefficients, and then finally determining the aggregate size of variance explained in the dependent variable by the proposed model. The mediation proposition was tested using the specific indirect effects provided on the model.

## Results

The Cronbach's alpha scores, as well as the composite reliability scores, confirmed the internal consistency of the scales as indicated in Table 1. The average variance extracted in and the heterotrait-monotrait scores, as well as the confirmatory factor analysis through the goodness-of-fit statistics, confirmed the distinctive, discriminant, and the convergent validity of technostress, perceived organisational support, work–life balance, and workplace flourishing. The composite reliability scores indicated in Table 2 observed a technostress scale Cronbach's alpha of 0.881, which is regarded as good (Pallant, 2020). The reliability scores associated with the dimensions of technostress were good, varying from 0.738 for techno-insecurity to 0.843 for techno-complexity. The internal consistency scores of work–family conflict dimensions were estimated, and the following scores were observed: time-based conflict, 0.845; strain-based conflict, 0.810; and behaviour-based conflict, 0.910, all considered as good. The perceived organisational support scale scored 0.900. The workplace flourishing scale was made up of three dimensions that all scored acceptable internal consistency scores (emotional wellbeing, 0.863; psychological wellbeing, 0.917; and subjective wellbeing, 0.935).

**Table 1:** Reliability of the scales.

**Variable**	**Number of** **items**	**Cronbach's alpha**
**Technostress**	21	0.881
Techno-invasion	4	0.773
Techno-overload	4	0.810
Techno-complexity	4	0.843
Techno-insecurity	5	0.738
Techno-uncertainty	4	0.810
**Perceived organisational support**	8	0.900
**Work–family conflict**	18	0.928
Time-based conflict	6	0.845
Strain-based conflict	6	0.810
Behaviour-based conflict	6	0.910
**Workplace flourishing**	21	0.957
Emotional wellbeing	3	0.863
Psychological wellbeing	9	0.917
Social wellbeing	5	0.935

**Table 2:** Quality criteria.

**Variable**	**Cronbach's**	**Composite**	**Average variance**
	**alpha**	**reliability**	**extracted**
Workplace flourishing	0.901	0.937	0.833
Work–family conflict	0.844	0.906	0.762
Technostress	0.736	0.802	0.512
POS	1.00	1.00	1.00

POS, perceived organisational support.

To determine model fit to the data, the following goodness-of-fit statistics were used: the root mean square error of approximation (RMSEA), the standardised root mean square residual (SRMR), and the comparative fit index (CFI). Little (2013) noted that, in most cases, models with RMSEA and SRMR lower than 0.05 and a CFI higher than 0.95 are regarded as representing a very good fit between the hypothesised model and the data. The measurement model for the three-dimensional model of workplace flourishing was stipulated through allowing each dimension to load on its respective latent factor (for example, the seven items representing psychological wellbeing, seven items reflecting emotional wellbeing, and another seven items for subjective wellbeing. A CFI of 0.891, RMSEA of 0.059, and SRMR of 0.051 were observed. The confirmatory factor analysis model fit indices related to technostress were observed as RMSEA = 0.052, SRMR = 0.063, and CFI = 0.956. Based on the results, the model fit the data well, since all the three fit statistics observed were statistically adequate. For the work–family conflict, the following fit statistics were discovered: SRMR = 0.0728, CFI = 0.940, and RMSEA = 0.123. The model can be considered to be adequate since two of the three fit statistics (SRMR and CFI) were acceptable.

### Quality criteria: Outer model

To assess the quality criteria the composite reliability was considered for the internal consistency reliability aspect. The results indicated that the composite reliability for all the variables was above the 0.6 cut-off score; therefore, it can be concluded that the four constructs in the study observed satisfactory composite reliability. The scores are as follows: workplace flourishing, 0.937; work–family conflict, 0.906; technostress, 0.736; and perceived organisational support (1.00). To assess convergent validity of the scales, the average variance extracted (AVE) score was applied and all observed as acceptable. All the AVE scores were above the 0.5 cut-off (work–family conflict, 0.762; workplace flourishing, 0.833; technostress, 0.512). Table 3 displays the findings for the discriminant validity, indicating the heterotrait-monotrait (HTMT) values observed for the variables: 0.179 for technostress and perceived organisational support, 0.414 for work–family conflict and perceived organisational support, 0.677 for work–family conflict and technostress, 0.695 for workplace flourishing and perceived organisational support (0.695), 0.218 for workplace flourishing with technostress, and 0.441 for workplace flourishing with work–family conflict. For a good discriminant validity, Hair et al. (2019) noted that the HTMT values should be lower than 0.90; thus, it is evident from the results that all the values obtained were lower than the cut-off. This enabled the study to proceed with the evaluation of the structural model, reflecting the proposed paths of the conceptual model. Table 4 shows the outer loadings, which are the paths linking each dimension or indicator to its relevant theoretical construct. From the table, it is clear that significant loadings were observed for all the indicators loading on their respective constructs with (*p* = 0.000). The loadings for the indicators were spread from 0.646 (techno-insecurity) to 0.930 (psychological wellbeing).

**Table 3:** Heterotrait-monotrait ratio discriminant validity.

**Variables**	**POS**	**Technostress**	**WFC**	**WF**
**POS**				
Technostress	0.179			
WFC	0.414	0.677		
WF	0.695	0.218	0.441	

POS, perceived organisational support; WFC, work-family conflict; WF, workplace flourishing.

**Table 4:** Indicator loadings: the outer model.

**Variable and dimension**	**Original sample (o)**	**Sample mean**	**Standard deviation**	***T*** **-statistics**	***P*** **-values**
Emotional wellbeing: workplace flourishing	0.899	0.898	0.014	62.168	0.000
Perceived organisational support	1.000	1.000	0.000		
Psych wellbeing: workplace flourishing	0.930	0.930	0.013	71.558	0.000
Social wellbeing: workplace flourishing	0.908	0.908	0.011	85.722	0.000
Techno-complexity: technostress	0.685	0.676	0.064	10.731	0.000
Techno-insecurity: technostress	0.646	0.638	0.063	10.275	0.000
Techno-invasion: technostress	0.832	0.832	0.030	28.125	0.000
Techno-overload: technostress	0.909	0.910	0.015	59.499	0.000
Strain-based: work–family conflict	0.889	0.887	0.020	43.734	0.000
Time-based: work–family conflict	0.875	0.874	0.018	48.299	0.000
Behaviour-based: work–family conflict	0.855	0.853	0.024	35.726	0.000

### Assessment of the measurement model

Table 5 indicates the path coefficients with the associated *p*- and *t*-values. The path coefficients provide an indication of the strength as well as the direction of the proposed theoretical paths. From the results, it is evident that all the proposed paths in the theoretical model are statistically significant at *p* < 0.05. The observed pathway from perceived organisational support to workplace flourishing was the strongest (β = 0.611: *t* = 12.40: mean = 0.611: *p* = 0.000). Technostress to work–family conflict observed the second strongest link (β = 0.524: *t* = 11.117: mean = 0.533: *p* = 0.000), implying that technostress is a strong determinant of work–family conflict. Perceived organisational support reported a negative but significant path to work-family conflict (β = −0.288: *t* = 5.003: mean = – 0.285: *p* = 0.000). Technostress to perceived organisational support also reported a negative but significant path (β = −0.178: *t* = 2.417: mean = −0.181: *p* = 0.016). Work–family conflict to workplace flourishing reported the least statistically significant path to the endogenous variable (β = −0.154: *t* = 2.635: mean = −0.155: *p* = 0.009). It is evident that two of the proposed paths to the dependent variable in the proposed theoretical model are statistically significant. A combination of all the independent constructs in the model explains ~47% variance in workplace flourishing. It is also clear that two of the three independent variables (perceived organisational support β = 0.611, *p* = 0.000 and work–life conflict β = −0.154, *p* = 0.009) observed significant direct relationships with workplace flourishing.

**Table 5:** Path coefficients: the inner model.

**Variables**	**Original sample (o)**	**Sample mean**	**Standard deviation**	***T*** **-statistics**	***P*** **-values**
POS—WLC	−0.288	−0.285	0.058	5.003	0.000
POS—WF	0.611	0.611	0.049	12.410	0.000
Technostress—POS	−0.178	−0.181	0.074	2.417	0.016
Technostress—WFC	0.524	0.533	0.047	11.117	0.000
WLC—WF	−0.154	−0.155	0.058	2.635	0.009

POS, perceived organisational support; WFC, work-family conflict; WF, workplace flourishing.

**Figure 1:**
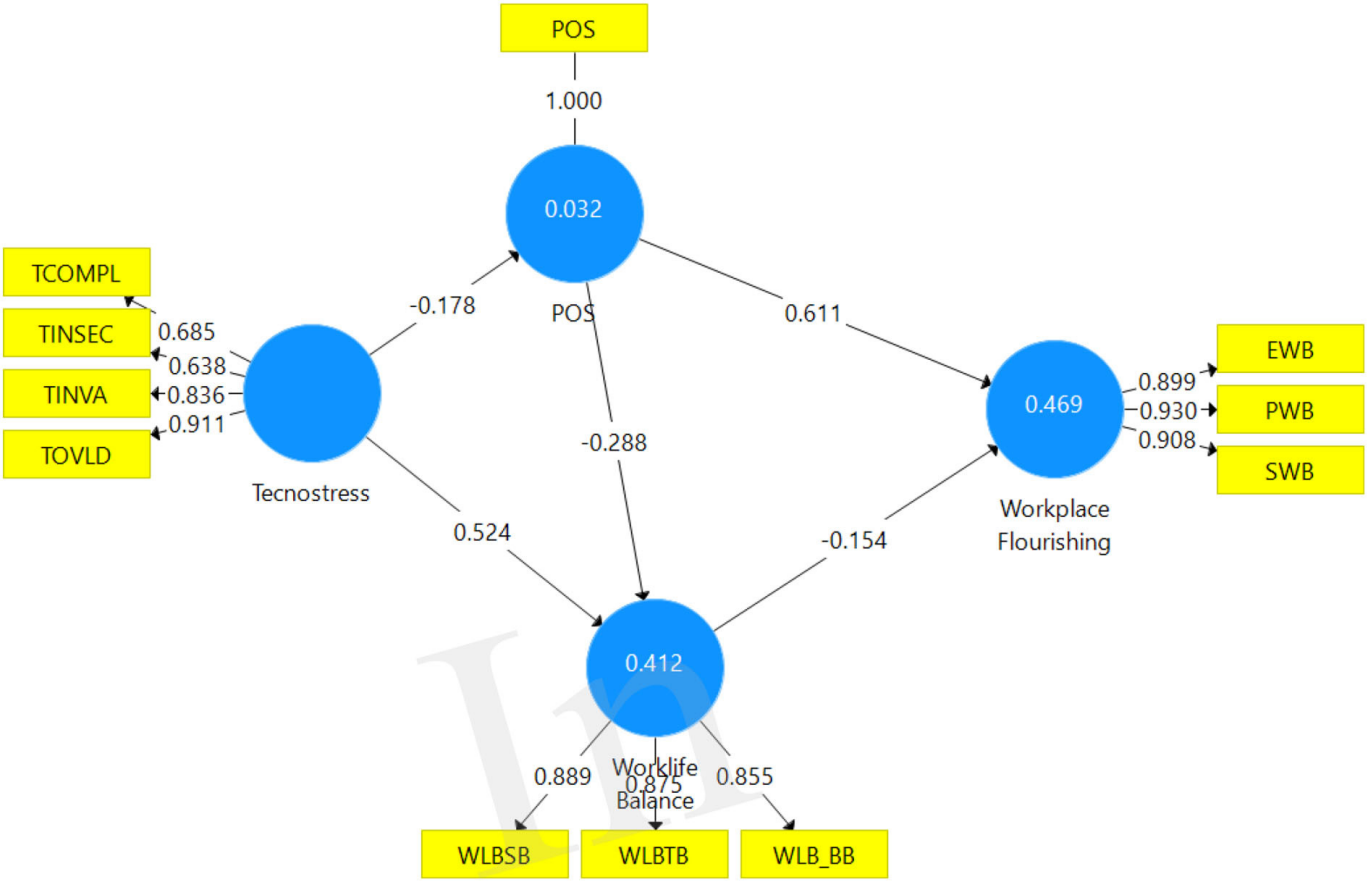
The model for the influence of technostress, work–family conflict, and perceived organisational support on workplace flourishing.

On the other hand, technostress had a non-significant direct relationship with workplace flourishing. These results, therefore, provide partial support for Proposition 1. Thus, work–life conflict and perceived organisational support have a direct influence on workplace flourishing. Table 6 shows the extent to which technostress, perceived organisational support, and work–family conflict influence workplace flourishing. The independent variables in the theoretical model (technostress, perceived organisational support, and work–life balance) explain ~46.9% = 47% of the variance in workplace flourishing, which, according to Chin (1998), is interpreted as moderate effect. Note is taken that perceived organisational support and work–family conflict both have a significant association with workplace flourishing. Note should also be taken that, whilst perceived organisational support exhibited positive significant influence on workplace flourishing, work–family conflict observed a negative statistically significant influence on workplace flourishing. The results provide partial support for Proposition 1. Figure 1 shows the significant paths from the independent variables to the dependent variable.

**Table 6:** R-squared.

**Variable**	**R-square**	**R-square adjusted**
POS	0.032	0.027
Work–family conflict	0.412	0.406
Workplace flourishing	0.469	0.464

POS, perceived organisational support.

To evaluate the other three propositions formulated, which relate to mediation, the indirect effects presented in Table 7 should be considered, examining if perceived organisational support mediates the relationship between technostress and workplace flourishing, and whether work–life conflict mediates the relationship between technostress and workplace flourishing, as well as whether both perceived organisational support and work–life conflict combined mediate the relationship between technostress and workplace flourishing. It is evident that work–family conflict significantly mediated (β = 0.044, *p* = 0.017) the relationship between perceived organisational support and workplace flourishing. It is also noted that perceived organisational support significantly mediated (β = −0.109, *p* = 0.021) the relationship between technostress and workplace flourishing. Work–family conflict mediated the relationship between technostress and work–life balance significantly (β = −0.081, *p* = 0.008). Therefore, the findings supported Propositions 3 and 2. However, the mediating effect for the ultimate path from technostress *via* POS through work–family conflict to workplace flourishing is not statistically significant (β = −0.008, *p* = 0.082); the mediating effect is smaller than that of perceived organisational support alone (0.109 vs. 0.008). Because the direct path coefficient between technostress and workplace flourishing is statistically insignificant, the results provide evidence of no direct influence between the two constructs.

**Table 7:** Specific indirect effects.

**Variables**	**Original**	**Standard**	***T*** **-statistics**	***P*** **-values**
	**sample (o)**	**deviation**		
POS–WFC–WF	0.044	0.018	2.429	0.017
Tech–WFC–WF	−0.081	0.032	2.533	0.008
Tech–POS–WFC	0.051	0.023	2.272	0.018
Tech–POS–WF	−0.109	0.047	2.326	0.021
Tech–POS–WFC–WF	−0.008	0.005	1.625	0.082

POS, perceived organisational support; WFC, work-family conflict; WF, workplace flourishing; Tech, technostress.

In addition, no significant mediating effect was observed between technostress and the combined perceived organisational support and work–family conflict on workplace flourishing. Therefore, the findings of the study found no support for Proposition 4, with both perceived organisational support and work–life conflict having an insignificant mediating effect (−0.008, *p* = 0.082) on the relationship between technostress and workplace flourishing.

## Discussion

The remote working settings imposed by COVID-19 caused significant and radical reconsideration of the nature of work within companies and institutions, which resulted in a shift in the way work is completed. The continuous use of ICT has resulted in strain leading to the unpleasant physiological activation that materialises in anxiety, tension, and discomfort (technostress), which has effects on employee health and wellbeing (Lund et al., 2020). The current study had a two-fold aim: to discover the direct and indirect influence of technostress, work–family conflict, and perceived organisational support on workplace flourishing in the context of remote working amidst COVID-19. It was proposed that technostress and work–family conflict negatively influence workplace flourishing (directly and indirectly), and perceived organisational support positively influences workplace flourishing.

Two of the three constructs had significant direct relationships with workplace flourishing. A positive relationship was observed between POS and workplace flourishing. These findings are consistent with the social exchange perspective on the employment relationship: Employees are more likely to flourish in their work when they perceive that their supervisors as well as the organisation at large are providing adequate support and are fulfilling their needs (Karim et al., 2019). Literature clearly indicates that POS is positively linked to psychological wellbeing (Caesens et al., 2016), which is part of the dimensions of workplace flourishing. This study is consistent with the research finding by Caesens et al. (2016), showing that POS is positively correlated with workplace flourishing. Thus, flourishing is intensified when crucial and significant resources related to a job such as POS are provided (Mahapatra and Pati, 2018). Consistent with that, the theory of conservation of resources (COR; Hobfoll, 2011) indicates that POS does not only establish the basis for the exchange relationship but also builds resources to maintain employee wellbeing (Panaccio and Vandenberghe, 2009). POS emerged as a strong predictor of workplace flourishing, and this relationship is not surprising, because De Paul and Bikos (2015) proved that the perception of a supportive organisation adds to improved outcomes of psychological wellbeing. Thus, POS is often equated to positive relations and support networks in an organisation, which result in workplace flourishing.

Part of the first proposition stated that perceived work–family conflict has a direct effect on workplace flourishing. The findings supported the proposition, indicating that work–family conflict negatively influences workplace flourishing. This implies that, when participants perceive a high experience of work–family conflict, they are more likely to exhibit low levels of workplace flourishing. The findings are consistent with Parris et al. (2008), Khan and Fazili (2016), and Gomes et al. (2021), who noted that perceived work–family conflict predicted workplace flourishing negatively, especially the psychological wellbeing dimension.

On the other hand, technostress was not significantly associated with workplace flourishing. This implies that the level of technostress of the participants did not positively or negatively influence their workplace flourishing directly. Contrary to these findings, based on the JD–R model, technostress is regarded as a job demand, which puts strain on employees and is expected to negatively influence workplace flourishing. Similarly, the stress-strain outcome (SSO) model by Cheung and Tang (2010) explains that a negative association between technostress creators and wellbeing does exist. The SSO model explains that, when exposed to technostress, users are likely to experience emotional strain, such as helplessness, anxiety, feelings of incompetence, and low confidence, which reflect languishing rather than flourishing. Although the findings are surprising and contradict the findings of other researchers (Salo et al., 2019; Tarafdar and Stich, 2021), who noted that employees who experience technostress may also experience burnout, poor psychological health, and even depression, it is important to note that the current study discovered indirect effects between technostress and workplace flourishing.

Consistently, although it has become difficult for individuals to complete most tasks and activities without incorporating technology, the technology poses a challenge of strain that may diminish flourishing (Janse van Rensburg et al., 2017). Although the use of ICTs has greatly enhanced the performance as well as the production efficiency, the adoption, and the diffusion of ICTs demand high social, cognitive, and physical skills, which have resulted in technostress. The implementation of the ever-changing technologies has resulted in more complex demands on jobs, which eventually affect work–life balance and wellbeing. The increased interdependency on ICTs demands a lot of effort and new knowledge from the employees, and this negatively influences workplace flourishing (Tarafdar and Stich, 2021).

In line with the above, some of the reasons why individuals languish at the hands of ICTs and fail to manage technostress and work–family conflict include lack of support during the installation, testing, and implementation of ICTs adopted by a company. In remote settings this may be fuelled by insecurity and discomfort, resulting from multitasking (Ragu-Nathan et al., 2008) and frequent interruption of assigned tasks due to the ongoing stream of communication (Mark et al., 2008). These stressors, coupled with a lack of personal coping mechanisms, result in low levels of psychological and emotional wellbeing. This is manifested through frustration, a sense of ineffectiveness, high mental load, time pressure (Mark et al., 2008), and reduction in work–life balance (Tarafdar and Stich, 2021), which eventually affects workplace flourishing. Thus, perceived technostressors can lead users to experience emotional problems and negatively affect social relationships and the general psychological wellbeing of employees. According to Janse van Rensburg et al. (2017), these aspects are of primary concern as they indirectly impact on workplace flourishing. Nevertheless, with adequate organisational support, the demands imposed by both technostress and work–family conflict are neutralised.

The second proposition noted that POS mediates the relationship between technostress and workplace flourishing. The proposition was supported. POS mediates the relationship between technostress and workplace flourishing. Thus, technostress influences workplace flourishing through perceived organisational support; those who experience technostress with high organisational support tend to flourish. These findings are consistent with other related empirical studies (e.g., Ujoatuonu et al., 2019; Springs, 2021), which indicated that POS may be considered a potent factor that, if properly instituted, may make the difference in the impact of technostress on flourishing. Consistently, with reference to the JD–R model (Bakker and Demerouti, 2014), job resources such as POS have the potential to buffer the negative effects of job demands such as technostress (techno-invasion, techno-complexity, and techno-overload), and job resources (POS) facilitate motivational processes, for example, an individual's desire for growth, which is regarded to be part of psychological and subjective wellbeing. This is the most important finding in this study, that there was no direct influence between technostress and workplace flourishing but a full mediation/strong indirect link between technostress and workplace flourishing through perceived organisational support.

The 3rd proposition noted that work–family conflict can mediate the relationship between technostress and workplace flourishing. This proposition was supported. Technostress had an indirect influence on workplace flourishing when work–family conflict was applied as a mediator; note is taken that the relationship direction is negative (β = −0.154). Accordingly, a negative significant test (*p* = 0.008) mediating effect of work–family conflict is confirmed in the relationship between technostress and workplace flourishing. Those who perceived low technostress tend to flourish when they have low work–family conflict. Thus, technostress through work–family conflict is negatively associated with workplace flourishing. This is consistent with the findings of other researchers (Brough et al., 2014; Casper et al., 2018; Powell et al., 2018), who noted that a few of the dimensions of technostress, including techno-invasion and techno-complexity, invade individual employees' family time and, in turn, negatively affect emotional and psychological wellbeing, which form part of workplace flourishing. The results are reasonable, considering that the intrusion of work into personal life caused by ICT intensifies the negative spillover between work and family and eventually influences flourishing. In remote settings, individuals experienced feelings of being always reachable and attuned to work issues without a break. Such experiences reflect the spillover of work technologies to the family time and result in conflict between work and family roles, which, eventually, negatively influence workplace flourishing.

According to Propositions 2 and 3, which were fully confirmed, POS mediates the relationship between technostress and workplace flourishing, and work–family conflict also mediates the relationship between technostress and workplace flourishing. However, the mediating effect of work–family conflict was weaker (−0.081) than that of technostress and perceived organisational support (−0.109). Technostress through work–family conflict had a negative influence on workplace flourishing. These findings are consistent with Mark et al. (2008), who discovered that it is hard to maintain the boundary between work and professional life with high levels of work–family conflict, coupled with techno-invasion, and hence difficult to experience workplace flourishing under those circumstances. This implies that, when technostressors are coupled with a lack of personal coping mechanisms that assist with balancing work and life demands, it becomes difficult for individuals to flourish. The technostress is manifested through frustration, a sense of ineffectiveness, high mental load, and time pressure (Mark et al., 2008), leading to a reduction in work–life balance (Tarafdar et al., 2007), which eventually affects psychological wellbeing (flourishing). It is, therefore, conceivable that, whilst experiencing technostress, employees respond better when they perceive that their organisation supports them and offers them security.

The fourth proposition, which proposed that perceived organisational support through work–life conflict mediates the relationship between technostress and workplace flourishing, was not supported. POS through work–family conflict does not mediate the relationship between technostress and workplace flourishing. These results are contrary to the COR theory, where work and family conflict and POS are considered to be reserves of job and personal resources, and the absence of resources in one domain influences the state of the other domain (Hobfoll, 2011). Both POS and work–family conflict are expected to impact personal burnout, distress symptoms, and employee wellbeing, which reflect workplace flourishing (Ibrahim, 2011; Fotiadis et al., 2019). Negative work–family interaction decreases workplace flourishing due to increased psychological strain and diminished mental resources (Voydanoff, 2002; Eby et al., 2005). The confidence of being in control over technology, work, and family activities is expected to have positive implications for workplace flourishing, specifically the psychological and emotional wellbeing components (Fotiadis et al., 2019).

In conclusion, the combined effect of technostress, POS, and work–family conflict on workplace flourishing indicated that POS is a critical component in the relationship between technostress and workplace flourishing. Putranto et al. (2021) noted that POS is seen as a job resource that lessens stress and supports and creates a feeling of security and satisfaction of employees' psychological and emotional needs for positive effect. Thus, despite the presence of technostress creators and work–family conflict issues, employees who regard their organisations as supportive can possibly experience positive psychological wellbeing and flourish rather than languish even when high demands are imposed on them. Highly flourishing individuals are more resilient toward life challenges and vulnerabilities, and such individuals benefit an organisation more since they are considered to be fit both physically and mentally compared to their languishing colleagues (Fotiadis et al., 2019). Within the JD–R model, individual technostress creators can act as job demands, and perceived organisational support acts as a resource that enhances workplace flourishing. Therefore, POS may assist individuals in coping with demands relating to technostress and conditions, leading to work–family conflict and thus enhance workplace flourishing.

## Practical implications

The utilisation of technology in the work context has several benefits for employees and for an organisation at large. However, as indicated in the findings, there are negative consequences, such as technostress and work–family conflict, that should be taken into consideration, and the necessary support should be provided. The current findings suggest that, since a negative relationship exists between technostress and perceived organisational support, in environments that are more prone to technostress and where workplace flourishing is threatened, managers need to maintain regular, transparent, and consistent communication to ensure that employees have adequate resources to deal with technology. When organisations provide adequate support for their employees in terms of ICT skills, even when exposed to technostress, this support creates positive results such as employee flourishing. Therefore, this suggests that fast and visible technical support during testing, implementation, and use of the ICTs adopted by the company is crucial.

In addition, supervisors should discourage certain behaviours that create technostress, such as multitasking, use of multiple devices, and use of real-time notifications. In line with that, to reduce the negative effects of techno-complexity, ICT leaders should create a culture of knowledge-sharing across a company and ensure that all employees are autonomously motivated to use the available ICTs (Al-Ansari and Alshare, 2019). Adequate support from managers is expected through recommending technical skills training for new devices, systems, and software; this should be coupled with the provision of adequate technical resources to integrate technology into daily work activities. To avoid boredom and information overload, consider making the ICT training more enjoyable, perhaps by making it game-based. Other interventions to support employees can include adequate forms of individualised ICT support that can be done over the phone, increased perceived organisational support through open communication, and employee valuation of the help received from ICT. In practice, ICT call-in services should be easily accessible to avoid techno-complexity and techno-uncertainty. Supervisors and technicians in organisations should make sure that there is accessible technical, emotional, physical, and mental health support for employees to ensure that individuals do not languish due to techno-complexity and techno-uncertainty in remote settings.

It should be noted that previous studies indicated that some of the attempts to inhibit technostress have been proved to be ineffective. More scientific approaches, such as positive technology, have been proved to be highly effective in reducing technostress and should be considered (Calvo and Peters, 2014). Positive technology is defined as a scientific and applied approach to the use of technology for improving the quality of our personal experience and making our work easier (Riva et al., 2012). This approach advocates that technology is used to generate positive experiences and is designed to support individuals in reaching self-actualising experiences, and it helps to improve connectedness between individuals or groups. Therefore, implementation of positive technology-designed solutions presents possible inhibitors of techno-overload, techno-complexity, and techno-invasion, and, in turn, increases workplace flourishing through autonomy and control. This eventually benefits individual wellbeing (Riva et al., 2012).

A positive relationship was also observed between technostress and work–family conflict, with the ultimate impact on workplace flourishing. Accordingly, when technostress and work–family conflict are high, low levels of flourishing are exhibited. These findings have implications for workplace flourishing in higher education. The managers need to realise the importance of helping both academic and support staff to flourish by instituting relevant organisational support and work–life balance policies that will help employees to flourish, especially when dealing with technostress creators in the remote work setting. To those struggling with work–family conflict, there is a dire need for managers to make allowances for employees to adjust their schedules to accommodate personal obligations, adjust employees' workloads to accommodate family responsibilities, and make it easier for employees to take paid time off. The struggle with both technostress and work–family conflict in the South African context is worsened by challenges regarding access to technology. Unique problems, such as load shedding, poor wi-fi connections, and the use of old devices, expose users to techno-unreliability strain, resulting in them spending too much time trying to complete tasks. These individuals will have less time and energy to devote to their family responsibilities. The study recommends that it is the responsibility of an organisation to ensure that each employee has access to strong wi-fi and devices that are compatible with the software and systems utilised by the organisation, and managers should set up policies to encourage employees to set boundaries, stop working, and switch off email notifications at the designated log-off time to maintain work–life balance and ensure psychological and emotional wellbeing.

Even though employees in remote settings are burdened by technostress, when they perceive positive work–life balance and adequate organisational support given by their supervisors, they tend to flourish, and flourishing eventually enhances performance. By developing favourable work–life balance policies, technostress creators, such as techno-invasion and techno-complexity, are controlled, and flourishing can be enhanced. This enhancement will, in turn, help the employees to actualise organisational and personal goals that give rise to institutional development and flourishing. The amount of technostress experienced and its effect may be enhanced or hampered by the prevailing atmosphere of work–life balance and the perceived organisational support received by employees. Therefore, POS and work–life balance will assist employees in enhancing their ability to flourish, deal with technostress creators, and balance work and family responsibilities. Furthermore, fostering flourishing through POS and work–life balance policies is a highly viable organisational goal that impacts important organisational outcomes.

Note is taken of the findings that the ultimate path from technostress through perceived organisational support *via* work–family conflict to workplace flourishing was found insignificant with no mediating effect. These unexpected results are consistent with a recent study (Lades et al., 2020), indicating that technology presumably allows more flexibility and autonomy, in turn resulting in employees working more and feeling in control, but, at the same time, although this improves the quality and accuracy of work, it interferes with family life, and potentially fosters expectations of permanent connectivity, which may be detrimental to workplace flourishing. Hence, the remote work context provides discordant results that can be explained in two ways. On the one hand, it enhances work–life balance and perceived autonomy; however, on the other hand, it has a negative impact on the quality of life, increases technostress, and calls for more organisational support. The above notion indicates how technology can possibly influence wellbeing, something which is required to enrich the field at the moment.

The research also detaches from the previous studies that focused mainly on observing the detrimental effect of technostress and work–family conflict on employees' wellbeing, and centred on the importance of the mediating effect of POS in the interaction between technostress and workplace flourishing in the remote work setting. The new findings of the study translate to practical implications for both employees and managers operating in remote settings, who are exposed to technostress and work–family conflict, suggesting that there is a need to increase organisational support as a way to positively influence workplace flourishing and lessen work–family conflict. It is highly recommended that leaders and supervisors play an active role in providing the required support for employees. This involves incorporating measures that reduce stress associated with the use of technology, specifically ensuring the provision of technical support, which has been proved to inhibit technostress (Li and Wang, 2021). Leaders have the responsibility to ensure that organisational demands on employees do not exceed normal working hours and normal workload, as the consequences for work–life balance and wellbeing are undebatable. Accordingly, supplemental work should be reduced or avoided, workload levels need to be constantly monitored by line supervisors, and communication during virtual work settings should be balanced, since an overload of emails can cause the development of technostress. Leaders should design proactive and family-friendly strategies to inhibit work–family conflict. Moreover, if organisations offer training and instruments to cope with the effects of technostress, the trainings should be short and interesting.

Success for both employees and organisations largely rest on the emotional, psychological, and subjective wellbeing of the employees, and for that reason it is crucial to cultivate a favourable work atmosphere that reduces technostress and work–family conflict, and to provide POS to stimulate employees to flourish rather than languish. In this regard, establishing the antecedents of flourishing and identifying its mediators are considered as the first step. Thus, understanding the mediating effect of work–family conflict and POS creates a platform for managers and organisations to enhance favourable conditions and strengthen positive mental states of employees. When individuals experience high POS, they feel secure, and they have adequate resources to control the technostress creators; thus, they are more effective and, eventually, they flourish.

### Limitations and future directions

Some limitations were identified for the study. Firstly, only four constructs were explored in the model, yet evidence from the literature notes that there are a number of other constructs that may also influence workplace flourishing. Future studies should thus consider including other positive variables, such as resilience, mindfulness, and work engagement. Secondly, data were collected in only one institution and focused mainly on the residential university employees. It should be noted that the effects of technostress and the type of support the institution offers may differ between a residential and an open distance e-learning (ODeL) institution. ODeL institutions, in principle, have implemented more ways of acting through ICT, and, therefore, their academics may experience less misfit (technostress) between the demands of the institution and their own needs regarding technology. In residential universities, these imbalances may be greater due to the lack of a tradition of fully integrating technology in teaching; accordingly, this restricts generalisation of results to residential institutions only. Although the study only includes a residential institution, the ODeL institutions may take the opportunity to replicate the study within their context. Further studies should consider focusing on more different institutions and larger samples to expand applicability of the findings in different situations. The third limitation was the utilisation of a cross-sectional self-report that, according to Podsakoff et al. (2012), involves possible method bias. Due to the cross-sectional survey method, it is also possible to lose sight of the impact of the timeline, especially on the workplace flourishing construct. Future studies could consider designing a follow-up survey to examine an overall perspective for assessing the effect of technostress on workplace flourishing. The study investigated technostress along with other variables; trying to examine technostress dynamics in particularly high-tech organisations would be helpful to identify and outline more contextualized interventions. In addition, it was difficult to completely establish whether the levels of workplace flourishing actually altered for the participants during the time they started remote working or whether it has remained constant. Although data were collected, with integrity and honest responses were gathered from the participants, it should be noted that the findings should be generalised with caution to the academic and support staff in South African institutions.

## Data availability statement

The raw data supporting the conclusions of this article will be made available by the authors, without undue reservation.

## Ethics Statement

The studies involving human participants were reviewed and approved by University of the Free State Economic Management Sciences Research Ethics Committee (UFS_GHREC) with reference number UFS-HSD2021/1827/21. The patients/participants provided their written informed consent to participate in this study.

## Author contributions

Both authors listed have made a substantial, direct, and intellectual contribution to the work and approved it for publication.

## Funding

The researchers funded the study on their own. The publication fees have been provided by the University of Pretoria and the University of the Free State.

## Conflict of interest

The authors declare that the research was conducted in the absence of any commercial or financial relationships that could be construed as a potential conflict of interest.

## Publisher's note

All claims expressed in this article are solely those of the authors and do not necessarily represent those of their affiliated organizations, or those of the publisher, the editors and the reviewers. Any product that may be evaluated in this article, or claim that may be made by its manufacturer, is not guaranteed or endorsed by the publisher.
